# Gamified family-based health exercise intervention to improve adherence to 24-h movement behaviors recommendations in children: “3, 2, 1 Move on Study”

**DOI:** 10.1186/s13063-023-07494-8

**Published:** 2023-08-14

**Authors:** Alicia M. Alonso-Martínez, Gaizka Legarra-Gorgoñon, Yesenia García-Alonso, Robinson Ramírez-Vélez, Loreto Alonso-Martínez, Blanca Erice-Echegaray, Mikel Izquierdo

**Affiliations:** 1 Navarrabiomed, Hospital Universitario de Navarra (HUN), Universidad Pública de Navarra (UPNA), Instituto de Investigación Sanitaria de Navarra (IdiSNA), Avenida de Barañain S/N 31008, Pamplona, Navarra Spain; 2 CIBER of Frailty and Healthy Aging (CIBERFES), Instituto de Salud Carlos III, Madrid, Spain

**Keywords:** Pre-school children, Family environment, Multicomponent exercise, Web app training, Adherence to exercise

## Abstract

**Background:**

Evidence suggests that movement patterns, including physical activity, sedentary behavior, and sleep duration, throughout a 24-h period, have a significant impact on biological processes and health outcomes for both young and adult populations. However, 80% of adolescents worldwide are not sufficiently active, and many children do not meet international physical activity recommendations for their age. Thus, the aim of this study is to evaluate the impact of a 12-week gamified family-based health and exercise intervention on physical fitness, basic motor competencies, mental and behavioral health, and adherence to 24-h movement guidelines in children aged 4 to 5 years old. The study will evaluate changes in sedentary levels, physical fitness, basic motor competencies, mental and behavioral disorders, adherence to the exercise program, and compliance with physical activity recommendations. In addition, the aim of this protocol is to describe the scientific rationale in detail and to provide information about the study procedures.

**Methods/design:**

A total of 80 children, aged 4 to 5 years old, will be randomly assigned in a 1:1 ratio to one of two groups: the exercise group and the routine care group. The exercise group will undergo a 12-week exercise intervention, followed by a 12-week follow-up period. On the other hand, the routine care group will undergo a 12-week period of routine care, followed by a 12-week follow-up control period. The exercise program will be implemented in a family setting and facilitated through a gamified web platform with online supervision, with the hypothesis that it will have a positive impact on physical fitness, anthropometric measures, basic motor competencies, and adherence to 24-h movement guidelines.

**Discussion:**

The results of this study will provide valuable insights into the impact of a gamified, family-oriented health and exercise program on various aspects of health, including physical fitness, basic motor competencies, mental and behavioral well-being, and adherence to 24-h movement guidelines. The findings will contribute to closing the gap in current knowledge on the effectiveness of these types of interventions for children and their parents. These findings will also contribute to  the development of future guidelines for promoting physical activity in children who do not meet the World Health Organization’s recommended levels. An online “3, 2, 1 Move on Study” is believed to increase accessibility, promoting health equity, and reducing economic barriers for all children and their families across diverse social groups.

**Trial registration:**

Trial registration: NCT05741879. Registered February 14, 2023, Version 1.

## Administrative information

Note: the numbers in curly brackets in this protocol refer to SPIRIT checklist item numbers. The order of the items has been modified to group similar items (see http://www.equator-network.org/reporting-guidelines/spirit-2013-statement-defining-standard-protocol-items-for-clinical-trials/).

**Table:** 

**Title** {1}	Gamified Family-based Health Exercise Intervention to Improve Adherence to 24-h Movement Behaviors Recommendations in Children: “3, 2, 1 Move on Study”
**Trial registration** {2a and 2b}	Clinicaltrial.gov: NCT05741879 https://clinicaltrials.gov/ct2/show/NCT05741879
**Protocol version** {3}	Version 1.1., February 14, 2023
**Funding** {4}	The “3, 2, 1 Move on Study” is supported in part by the Health Department of Gobierno de Navarra, and 50% co-financed by the European Regional Development Fund (ERDF) under Operational Programme 2014–2020 in Navarre
**Author details** {5a}	1. Navarrabiomed, Hospital Universitario de Navarra (HUN), Universidad Pública de Navarra (UPNA), Instituto de Investigación Sanitaria de Navarra (IdiSNA), Pamplona, España 2. CIBER of Frailty and Healthy Aging (CIBERFES), Instituto de Salud Carlos III, Madrid, Spain
**Name and contact information for the trial sponsor** {5b}	Gobierno de Navarra. Sección de Investigación, Innovación y Gestión del Conocimiento, Pabellón de Docencia, HUN, C/ Irunlarrea, 3. Pamplona, Phone: 848 422,623 / 848 422,810, E-mail: investigacion.salud@navarra.es
**Role of sponsor and funder** {5c}	Health Department of Gobierno de Navarra and ERDF played no part in study design; collection, management, analysis, and interpretation of data; writing of the report; or the decision to submit the report for publication

## Introduction

### Background and rationale {6a}

Early childhood is a crucial period for the development of physical, social, and cognitive abilities and the formation of healthy lifestyle habits that can extend into adulthood [1–3]. Unfortunately, physical inactivity is one of the leading causes of mortality worldwide, contributing to numerous health issues such as weight gain, obesity, and non-communicable diseases such as breast and colon cancer, diabetes, and heart disease [4]. There is strong evidence that the pattern of movement behaviors, including physical activity, sedentary behavior, and sleep duration, throughout a 24-h period affects biological processes and can impact health outcomes in both young and adult populations [5–7]. Regular physical activity has been shown to prevent and manage non-communicable diseases [8]. Unfortunately, 80% of adolescents globally are not sufficiently active [9], and many children do not meet international physical activity recommendations for their age [10]. In 2016, the Foundation for Nutritional Research, Physical Activity in Children and Adolescents reported that half of the population in Spain did not meet these recommendations [11]. The decline in physical activity between the ages of 11 and 15 is a concerning trend in most European countries, including Spain, with a more pronounced drop in girls (over 60%) [12]. It is important to address this issue to promote healthy physical and mental development in children.

In 2019, the World Health Organization (WHO), at the request of the Commission to End Childhood Obesity published the recommendations for physical activity, sedentary behavior, and screen and sleep time for children under the age of 5. According to these guidelines, children aged 3 to 5 years should engage in at least 180 min of physical activity of any intensity each day, including at least 60 min of moderate-to-vigorous-intensity physical activity [13]. Despite the numerous benefits of physical activity for young children, many preschoolers do not meet these recommendations [14]. A study conducted in Spanish children and adolescents aged 3 to 16 years old found a decline in physical activity levels and an increase in both screen and sleep time [15]. The authors of the study noted that preschool children aged 3 to 4 years old reduced their total PA by 92 min per day and increased their screen time by 2.2 h per day. The WHO recognizes the need for further high-quality studies that examine the full 24-h day of physical activity and a broader range of health indicators such as motor, cognitive, and psychosocial development and the long-term effects of early interventions. Additionally, studies that consider confounders such as diet and that establish standardized procedures and objective measurements to enable comparison between studies are also needed [16].

Physical activity plays a crucial role in the growth and development of preschool children, providing both immediate and long-term benefits for physical and psychological well-being [17, 18]. A recent systematic review demonstrates a positive association between physical activity and brain structure in various regions of the brain [19]. There is a significant correlation between brain structure and cognitive functions, indicating that a healthy brain structure can improve human functionality [19]. Another meta-analysis addresses the impact of physical exercise on executive functions in preadolescents children, adolescents, and young adults, concluding that short periods of high-intensity physical exercise lead to immediate improvements in executive functions. However, there is limited research on the effect of long-term exercise programs on academic and cognitive performance [20]. Additionally, the evidence for this age group is still relatively small compared to that of children and teenagers between 6 and 17. Currently, there is no evidence on the cost-effectiveness of these interventions in children under 5 years of age and their parent(s) [13].

### Objectives {7}

The objective of this study is to evaluate the impact of a 12-week gamified family-based health and exercise intervention through a gamified web platform with online supervision on physical fitness, basic motor competencies, mental and behavioral health, and adherence to 24-h movement guidelines in children aged 4 to 5 years. It is based on the global recommendations for 24-h movement [13] and focuses on promoting behavior change through family and community-level interventions without the need for additional equipment or facilities. The study will utilize a combination of anthropometric tests, objective techniques such as accelerometry, and assessments of physical fitness and motor competencies, along with questionnaires on eating habits to address confounding factors [21–23].

We propose a hypothesis that a 12-week physical exercise and lifestyle improvement program, implemented in a family setting and facilitated through a gamified web platform with online supervision, will have a positive impact on physical fitness, basic motor competencies, and adherence to 24-h movement guidelines in children aged 4 to 5 years old.

### Trial design {8}

The “3, 2, 1 Move on Study” trial will be a monocentric, single-blind parallel-group randomized controlled trial. All items from the ClinicalTrials.gov registry platform can be found within the protocol. The protocol will be developed according to the Standard Protocol Items: Recommendations for Interventional Trials (SPIRIT) guidelines for randomized controlled trial (RCTs) [24].

## Methods: participants, interventions, and outcomes

### Study design {9}

This pragmatic trial is currently taking place at “Iturrama Primary Care” (Pamplona, Navarra, Spain). This site has participated in previous clinical trials. For more details, refer to the Clinical Trials Registry: NCT05741879. The site is supported by associate investigator (AI) pediatrician leaders, who provide ongoing clinical expertise and support for the study. We will recruit eligible participants from general pediatrician wards at site.

### Eligibility criteria {10} and recruitment {15}

A total of 80 children of both sexes between the ages of 4 to 5 years old will be recruited from the “Iturrama Primary Care” (Pamplona, Navarra, Spain). Participants will be admitted if their parent(s)/guardian(s) have provided informed consent for their participation in the project and if, according to their pediatrician, they do not have physical or mental disorders that would prevent them from completing the assessment tests or participating in the exercises provided in the intervention program. It is a requirement to have at least one parent/guardian answer the questionnaires included in the protocol. Participants (children) who at the start of the project present a psychiatric disorder or chronic illness that limits their participation in physical activities, those who do not comply with the established procedures, and those who do not understand the Spanish language will be excluded from the study.

### Who will take informed consent {26a}

Throughout this protocol, the trial’s staff is responsible for weekly monitoring of various aspects, including regular reporting on informed consent. Prior to participating in the study, all parents or legal guardians will be required to sign an informed consent form. An email will be sent to them outlining the study’s objectives and procedures and encouraging them to ask any questions they may have. Additionally, it is advisable to seek the child’s perspective on the matter.

### Additional consent provisions for collection and use of participant data and biological specimens {26b}

On the consent form, participants will be asked if they agree to use of their data should they choose to withdraw from the trial. Participants will also be asked for permission for the research team to share relevant data with people from the universities taking part in the research or from regulatory authorities, where relevant. This trial does not involve collecting biological specimens for storage.

## Interventions

### Explanation for the choice of comparators {6b}

The comparator is routine care as per “Iturrama Primary Care” procedures informed by the WHO international practice guidelines [13].

### Intervention descriptions {11a}

**Fig. 1:**
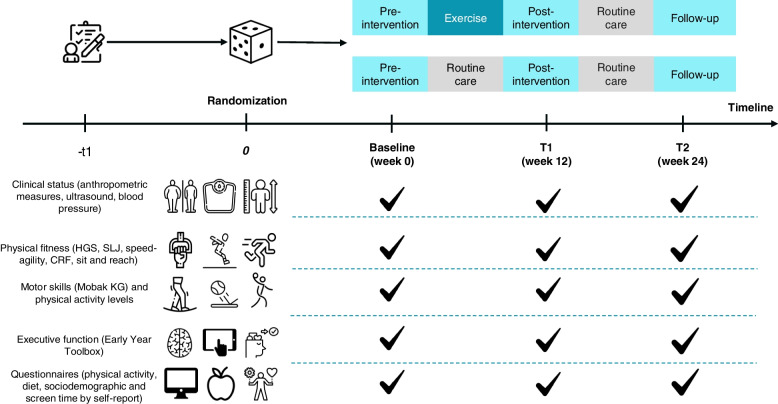
Flow of subjects through the “3, 2, 1 Move on Study.” Participants will be first randomly assigned to a 12-week period of standard daily routines (*n* = 40) or exercise training (*n* = 40). Both groups will be included in a 12-week follow-up period

Participants will be randomly assigned in a 1:1 ratio to one of two groups: the exercise group and the routine care group. The exercise group will undergo a 12-week exercise intervention, followed by a 12-week follow-up period. On the other hand, the routine care group will undergo a 12-week period of routine care, followed by a 12-week follow-up period. If participants are not engaging 2 times per week, the research team will reach out through email and/or phone to get a clear explanation as to why and to motivate the participants to continue. If the participants do not perform 70% of the training (or 17 out of 24 sessions), they will not be allowed to continue participation. The overall study procedure is illustrated in Fig. 1.

### Exercise group

**Fig. 2:**
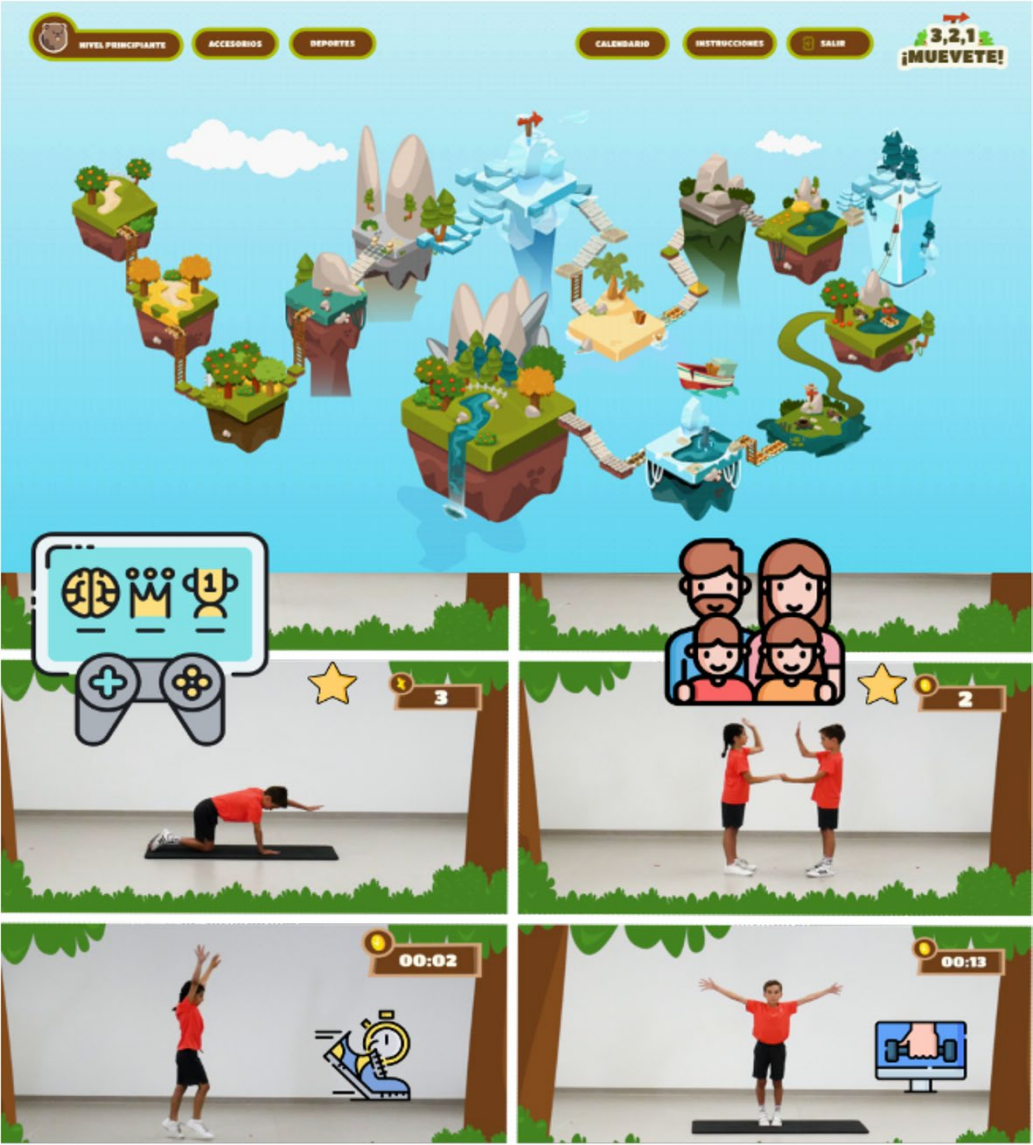
Example of gamified online platform

If allocated to the intervention group, participants will undergo a 12-week exercise intervention utilizing a gamified online platform designed for children to engage in physical activity with their families at home or in outdoor spaces (Fig. 2), followed by a 12-week follow-up period. The platform features pre-recorded video exercises and is designed to be followed by normal daily routines. Participants are required to log in and complete the exercises 2 times per week, with a different session available Monday to Wednesday and repeated from Thursday to Sunday. The program consists of various phases, including bodyweight strength training, cardiovascular exercises, and color-coded exercises, with varying exercises and repetition counts or duration from week to week. Each session includes a warm-up/activation phase, a training phase, and a cool down phase. The warm-up phase is 3 min of light intensity continuous training, the training phase is 10–26 min with 6–12 repetitions, 20–40 s of work, and 10–20 s of recovery, and the cool down phase is 3 min of static and dynamic exercises. The exercises will change each week, increasing in length and intensity. The program will be supervised by the child’s parent(s) or guardian(s).

### Routine care group

Participants allocated to the control group will continue to receive routine care according to WHO international practice guidelines [13], followed by a 12-week follow-up period. These activities include engaging in physical education class at school and/or playing with friends at the park or in the backyard. To ensure adherence to the intervention, the research team will closely monitor participant engagement, reaching out to participants who are not logging in twice per week to understand the reasons for non-compliance and to provide motivation to continue. Participants who do not execute their training will not be allowed to continue with the study.

## Criteria for discontinuing or modifying allocated interventions {11b}

### During intervention

Several measures will be taken in order to protect the participants’, child’s parent(s), or guardian(s) wellbeing and identity as follows. Firstly, according to the exclusion criteria, participants suffering from medical conditions or other serious health conditions that are incompatible with undertaking an exercise regimen will not be considered for inclusion and will immediately be referred to healthcare professionals. Secondly, if participants’ physical or emotional condition deteriorates during the trial, they will immediately be excluded from the trial and will similarly be referred to appropriate practitioners. Finally, participants will be informed that participation is voluntary and that they may discontinue the intervention at their free will. Any adverse events and other unintended effects of trial interventions or trial conduct will be addressed by the project coordinator (IRP).

### Strategies to improve adherence to interventions {11c}

The “3, 2, 1 Move on Study” research staff received training regarding Good Clinical Practice (GCP), the study protocol (including subsequent amendments), intervention, recruitment strategies, data collection using an electronic database, and inspection. A manual detailing the standard operating procedures (SOP) for the trial (including subsequent updates) will be available to all members of the research team. These will ensure ongoing protocol adherence and consistency. The information provided ensures all participants receive identical verbal and written information and instructions regarding the study. Regular assessment of treatment fidelity by the clinical trial coordinator (CTC) focuses on data integrity, the trial compliance, and its application.

### Relevant concomitant care permitted or prohibited during the trial {11d}

Routine care according to hospital procedures and informed by state governance and international standards (12) continues throughout the trial for all participants.

### Provisions for post-trial care {30}

Routine care according to hospital procedures and informed by state governance and international standards (12) is provided within the public health service in Navarra, Pamplona (Spain).

There is no anticipated harm and compensation for trial participation. There will be no provisions for post-trial care.

### Patient and public involvement (PPI)

To incorporate PPI, we will establish a collaboration with a local pediatric patient advocacy group and engage with parents, caregivers, and children themselves. Through focus group discussions, surveys, and one-on-one interviews, we sought their insights on the study objectives, exercise interventions, outcome measures, and participant experience.

## Outcomes {12}

### Primary outcome

The primary outcome will be mean minutes of moderate-to-vigorous physical activity (MVPA) per day assessed using GENEActiv accelerometers. The study will be able to detect a difference in the mean daily MVPA between intervention and routine care of ± 5 min at follow-up.

### Secondary outcomes

#### Anthropometric and body composition assessment

Participants will undergo testing while wearing minimal clothing and without footwear. Anthropometric measurements will be taken to determine weight, height (Seca 213, Seca Ltd, Birmingham, UK), waist circumference (WC), and body mass index (BMI). These values will be compared against WHO standards [25]. Additionally, body composition will be evaluated to determine the percentage of fat and lean mass, using bioelectrical impedance analysis (Tanita DC430 MA, Tanita Corporation, Tokyo, Japan), and ultrasound scans from the femoral rectus for muscle thickness, subcutaneous adipose tissue (SAT), and area of the muscle of interest. In addition, the medical team involved in the project will assess children’s systolic and diastolic blood pressure and triceps skinfold thickness.

#### Health-related physical fitness

Data on specific physical fitness related to health will be obtained with the 20-m shuttle run test (adapted to the children’s age) to assess aerobic capacity, the handgrip strength test (Grip Strength Dynamometer T.K.K. 5001 Grip A) and standing long jump test to assess musculoskeletal capacity, the 4 × 10-m speed and agility test to assess motor capacity, and the sit and reach flexibility test to measure trunk flexibility.

In Spain, the PREFIT battery is being administered [26] to provide national physical fitness reference values that interpret the results of the physical fitness assessment. Physical fitness, especially cardiorespiratory capacity and muscular strength, is a good indicator of health in children and adolescents [27]. A review of 32 studies found that the 20-m shuttle run test, the handgrip strength test, the standing  long jump test, the 4 × 10-m shuttle run agility test, and the BMI, skinfold, and waist circumference tests are reliable field-based fitness tests for children and adolescents [28].

#### Physical activity and sedentary habits

Data on physical activity levels will be collected using accelerometry over 7 consecutive days on the non-dominant wrist to record physical activity and sleep information (GENEActiv), using the YAP-S (Youth Activity Profile-Spain) questionnaire adapted for preschoolers to evaluate physical activity and sedentary habits, and the GPAQ (Global Physical Activity Questionnaire) to evaluate the parent’s physical activity.

#### Basic motor competencies (BMC)

The assessment of basic motor competencies (BMC) in preschoolers aged 4 to 6 years was conducted using the MOBAK KG test instrument [29]. The test consists of 8 items, divided into 4 object movement (OM) competencies and 4 self-movement (SM) qualifications. OM tasks include throwing, catching, bouncing, and dribbling, while SM tasks include balancing, rolling, jumping, and running. Children can earn a maximum of 8 points per area (4 items, 2 points each), totaling 16 points.

Before performing the tasks, participants were not given any prior attempts to practice. For the “throwing” and “catching” tasks, participants had six attempts to complete the task. Scoring was based on the number of successful attempts, with 0–2 attempts scoring 0 points, 3–4 attempts scoring 1 point, and 5–6 attempts scoring 2 points. In the “bouncing,” “dribbling,” “balancing,” “rolling,” “jumping,” and “running” tasks, participants had two attempts to complete each task. These items were scored dichotomously (0 = failed; 1 = passed), with the number of successful attempts recorded. A score of 0 points was awarded for never passed, 1 point for once passed, and 2 points for passed twice.

BMC play a vital role in child development and are a primary focus of physical education. Children aged 3–6 years are at a crucial stage in improving their motor skills, as everything they do involves motor actions such as posture, movement, exploration, social interaction, and object manipulation [30]. A validated and simple tool for assessing BMC is the MOBAK KG test, which evaluates two competence areas: object movement (OM) and self-movement (SM) [31].

#### Executive function

Participants will be assessed for their executive function using digital test on iPads, administered by research assistants. These tests will include games from the “Early Tools” (Years Toolbox YET-2017) such as “Mr. Ant” and “Not this” to measure memory, “Card sorting” to evaluate cognitive flexibility, and “Go/No-Go” to assess inhibitory capacity.

#### Quality of life

Data on the quality of life and self-regulation abilities of the children will be assessed using the KIDDY-KINDLR and the Child Self-Regulation & Behaviour Questionnaire (CSBQ) [32]. The KIDDY-KINDLR will assess physical and psychological well-being, self-esteem, family, friends, and school. The following six dimensions of children’s quality of life will be assessed through questions in a standardized questionnaire: physical well-being, psychological well-being, self-esteem, family, friends, and school. Participants will be asked about their experiences in each of these areas over the past week. Questions may include the following: “Have you felt sick?” for physical well-being, “Have you laughed and had lots of fun?” for psychological well-being, “Are you proud of yourself?” for self-esteem, “Have you gotten along with your parents?” for family, “Have you played with your friends?” for friends, and “Have you been able to do your homework well?” for school.

#### Cognitive and emotional self-regulation

For children over 5 years old, the Skills and Difficulties Questionnaire (SDQ) will be used to assess cognitive and emotional self-regulation, behavior problems, emotional symptoms, hyperactivity/attention deficit, peer relationships, prosocial behavior, and externalizing and internalizing problems. International studies have shown that the SDQ has sufficient validity as a detection and screening tool for children and adolescents [33]. In Spain, normative values for the Spanish version of the SDQ have been available since 2014 [34]. Therefore, the SDQ is a valuable tool for measuring and detecting emotional and behavioral problems in children. In addition, a standardized Child Self-Regulation and Behavior Questionnaire (CSBQ) will be utilized to assess cognitive and emotional self-regulation, sociability, prosocial behavior, and the presence of externalizing and internalizing problems in children. The questionnaire will be completed by the participants’ parent(s)/guardian(s) and will be collected through Google Forms.

#### The Youth Activity Profile-Spain (YAP-S) questionnaire

The following data will be collected using a standardized questionnaire through Google forms, completed by the participants’ parent(s) or guardian(s), to assess the children’s physical activity levels [35]. The questionnaire has been adapted to meet the needs of preschoolers aged 4 to 5 years old and includes additional questions, such as “Does your child enjoy participating in basic motor competencies classes offered at school?” “How many sports and/or cultural activities does your child participate in per week?” “Does your child have a television in their room?” “Is there a limit on the time your child is allowed to spend using digital devices, such as television, video games, computers, and phones?” “Are there any green areas or sports centers in your neighborhood? and Has a doctor recommended physical activity for your child in the past 12 months?”.

#### Mediterranean Diet—Children’s Eating Habits Questionnaire

The following dietary information will be recorded in a standardized questionnaire through Google forms, completed by the child’s parent(s) or guardian(s) [36]. For beneficial components (for example, vegetables, legumes, fruits, cereals, fish, dairy products, olive oil), the frequency scoring is higher than for components considered less beneficial (meat, fast food, confectionery).

#### Sociodemographic questionnaire

Variables measured by self-report will be collected via a standardized questionnaire completed by the participants’ parent(s)/guardian(s) using Google forms, such as child’s information (e.g., school, gender, age, place of birth, ethnicity, language spoken at home, and languages spoken by the child), and parent(s)/guardian(s) information (e.g., place of birth, age, highest education level, highest professional qualifications, employment status, job position, and monthly income, and household location and neighborhood).

### Participant timeline {13}

**Fig. 3:**
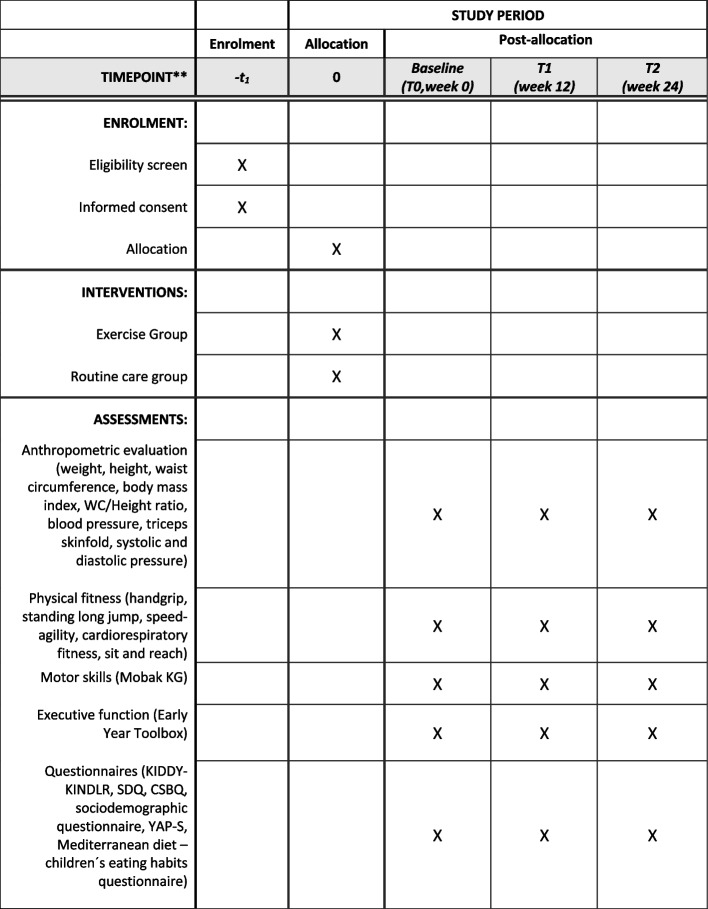
Schedule illustrating enrolment and interventions (SPIRIT figure) of the “3, 2, 1 Move on Study”. T, time-point. According to SPIRIT 2013 statement. Double asterisks (**) indicate the following: a random subsample of participants (40 subjects in each group, *n* = 80)

The outcome measures will be assessed at four time points [at baseline, at week 0 (T0), week 12 (T1), and week 24 (T2)], and the parent(s)/guardian(s) will be reminded to complete the questionnaires within a week. If needed, the research assistants will schedule an interview to complete the questionnaires on a tablet (Fig. 3).

### Sample size {14}

There was some evidence to suggest that physical activity interventions [37] led to an improvement in the proportion of children who engaged in MVPA during scholar day (odds ratio (OR) 2.74, 95% confidence interval (CI), 2.01 to 3.75). Study parameters are as follows: alpha = 0.05, power = 0.80, delta =  − 1.37 (OR difference with control group). After adjustment for an estimated 10% estimated dropout rate (a similar rate has been observed in previous systematic reviews [38]), 42 participants per group will be needed for sufficient power (difference in the mean daily MVPA between groups of ± 5 min) at follow-up.

## Assignment of interventions: allocation

### Sequence generation {16a}

Eligible participants will be randomly assigned to one of two groups using a computer-generated block randomization schedule (Research Randomizer V.4). Assignment will take place through previously constructed balanced blocks, which will allow equiprobable assignment and ensure that each group has the same number of participants. This allocation mechanism will be carried out until 84 total participants are assigned.

### Concealment mechanism {16b}

Randomization will computer-generated via a secure website and can be accessed via an electronic device.

### Implementation {16c}

Participants will log in to a centralized monitoring service on the Navarrabiomed site through a secure website using a mobile electronic device. This process ensures that the trial is independent and secure.

## Assignment of interventions: blinding

### Who will be blinded {17a}

The assignment password will be kept in a confidential file in the Navarrabiomed center and will be opened at the end of the study’s analyses. To ensure masking, an alpha-numerical code will be assigned to each study group. This code will be delivered to the associated researcher and will not be revealed to the investigators in charge of processing the data until the analysis of the coded interventions is completed. Additionally, the researchers from the Navarrabiomed center will be responsible for preparing reports for the participants’ safety committee to verify the progress of the study. Following the conclusion of the baseline examination, the appropriate envelope will be opened by a study researcher, and the participants will be informed about their group allocation. Assessors of outcomes will be blinded to patient data, including allocation at baseline and follow-up. Blinding will be used for all specialized analyses. Owing to the nature of the study, patients cannot be blinded to the exercise training modality.

### Procedure for unblinding if needed {17b}

The design is open label with only outcome assessors being blinded, so unblinding will not occur.

### Data collection and management

#### Plans for assessment and collection of outcomes {18a}

The research staff will collect data on site, using questionnaires on secured Google Forms™ which will be assimilated in a password-protected Google drive™. All the parameters assessed in the study are defined a priori in a data dictionary elucidating standards for data collection.

#### Plans to promote participant retention and complete follow-up {18b}

Retention will be calculated as the number of participants who complete the entire 12-week training program divided by the total number of participants, also expressed as a percentage. A minimum adherence rate of 70% is considered satisfactory. Any data analysis disagreements will be resolved by a third-party biostatistician.

### Data management {19}

Data will be entered directly into access database, via a secure web platform for building and managing online databases, by Navarrabiomed using a password-protected computer. All data retained will be in an electronic, re-identifiable format. Paper-based/non-electronic data (e.g., data collection instruments) will be transferred to an electronic format and all paper-based/non-electronic data will be destroyed.

### Confidentiality {27}

All participants will be assigned a study-specific identification number. Exported data will be de-identified and stored separately from the dataset containing participant identifying information (only the participant’s unique study number is contained in both files for the purpose of re-identification). Any study information will be stored on a password-protected computer or secure Navarrabiomed applications accessible only by the research team.

### Plans for collection, laboratory evaluation, and storage of biological specimens for genetic or molecular analysis in this trial/future use {33}

No biological specimens will be collected as part of this trial.

## Statistical methods

### Statistical methods for primary and secondary outcomes {20a}

The study will adhere to the intention-to-treat principle and use the baseline observation carried forward imputation method, where missing data will be replaced by baseline values [39]. The final analysis population will be described and any differences from the enrolled population will be reported. Participants who drop out of the study may still be included in the analysis, although their data will provide less information and lower the estimated effects variance. Adherence will be measured as the number of completed sessions divided by the total number of sessions, expressed as a percentage. The first step of the analysis will be to calculate descriptive statistics for the overall sample and between groups. Since this is an experimental design with primary and secondary outcomes measured at three timepoints - baseline (t0 = week 0), post-intervention (t1 = week 12), and after a 12-week follow-up period (t2 = week 24) - for the two study groups (exercise and routine care), a comparative analysis between the measurements will determine the differences. A linear mixed model with repeated observations will be used for these comparisons, when appropriate, for each dependent variable. Subsequently, multivariate analysis will be carried out, and the autocorrelation between repeated measures will be taken into account. We will use longitudinal analysis methods, such as a generalized estimating equation approach, to control the differences among measurements at baseline and to incorporate incomplete observations into this analysis. Pearson correlations will be calculated between the maximal values of the primary and secondary outcomes. All analyses will be 2-sided and a significance level of *α* ≤ 0.05 will be used for the primary analysis.

### Interim analyses {21b}

Based on the study design, sample size calculation, participant safety, ethical considerations, and resource allocation, it has been determined that the trial will not incorporate interim analyses or formal stopping rules. The decision aims to optimize the study’s scientific integrity, participant safety, and resource utilization while ensuring that the trial yields reliable and conclusive results upon its completion.

### Methods for additional analyses (e.g., subgroup analyses) {20b}

Subgroup analysis will be conducted to understand if a 12-week PA program will generate adherence to the practice of physical activity and thus help the children meet the recommendations for physical activity, restful sleep, and sedentary habits is more or less effective than standard daily routine. All analyses will be performed using commercially available statistical software.

### Methods in analysis to handle protocol non-adherence and any statistical methods to handle missing data {20c}

The potential effects of missing data will be explored under various models for nonignorable missing data mechanisms and through multiple imputation models under ignorable missing data assumptions [40].

### Plans to give access to the full protocol, participant-level data, and statistical code {31c}

The study is registered on ClinicalTrials.gov (NCT05741879). A complete, cleaned, de-identified copy of the final dataset used in conduction of the final analyses will be made available within 1 year of study completion to protect the identity of interviewees. Outside investigators will be required to follow all the protocols for confidentiality, security, notifications to the study sponsor (e.g., Health Department of Gobierno de Navarra and ERDF), acknowledgments of funding, etc., required of the research team.

## Oversight and monitoring

### Composition of the coordinating center and trial steering committee {5d}

The trial will be supported by a dedicated coordinating center from Navarrabiomed that oversees the day-to-day operations and provides essential organizational support. The coordinating center comprises a team of experienced research professionals, including project managers, data managers, statisticians, and administrative staff. The research team is composed of experts from various health and physical activity sciences, as well as researchers from the Public University of Navarra and Navarrabiomed. The project will also involve pediatricians, pediatric nurses, a family doctor, and family and community medicine MIRs from the Iturrama Primary Care center. Standardization in data collection will be ensured through training and coaching. The team will monitor and report adverse events, with prompt reporting of severe adverse events to the designated committee in accordance with recommendations. The team will also track recruitment and follow-up, participant adherence, and quality control. The monitors will be available through a designated phone number and email address.

### Composition of the data monitoring committee, its role, and reporting structure {21a}

The team will monitor and report adverse events, with prompt reporting of severe adverse events to the designated committee in accordance with recommendations.

A formal data monitoring committee will not needed for the current trial. This is because the participants will not be exposed to significant harms and because of its short duration.

### Adverse event reporting and harms {22}

Serious adverse events associated with the intervention will be reported to the Iturrama Primary Care Center and human research ethics committees from the Public University of Navarra.

### Frequency and plans for auditing trial conduct {23}

As outlined throughout this protocol, the staff will monitor all aspects of the trial on a weekly basis. This includes adherence to the protocol, ethics and governance, management of databases, outcomes assessments, training of research staff, and regular reporting to informed consent. The intervention employed in this trial has been extensively studied and has a well-established safety profile. The potential risks associated with the intervention are minimal, and there is no anticipated harm that would necessitate early termination based on safety concerns. The data monitoring committee was not considered as this was a low-risk intervention.

### Plans for communicating important protocol amendments to relevant parties (e.g., trial participants, ethical committees) {25}

Protocol amendments will be approved of by the human research ethics committees prior to implementation. If relevant, current participants will be informed of protocol modifications. The ClinicalTrials.gov registry for this study will be updated with important protocol amendments.

### Dissemination plans {31a}

Results will be presented in aggregate form to ensure personal information is non-identifiable. We will present results at the local hospitals and recommendations for future research for dissemination to various media and professional groups. Conference abstracts will be submitted to major international meetings of wound prevention and treatment, nursing, and medical groups. Trial results will be published in high-impact generalist journals. Aggregated results will also be given to participants upon request.

## Discussion

The current study aims to examine the effects of a 12-week multicomponent and supervised physical exercise program on body composition, health-related physical fitness, physical activity, sedentary habits, and executive function in 4- to 5-year-old children. The study seeks to address the current lack of physical activity among this age group, as it is estimated that most children in this age range do not meet the recommended 180 min of daily physical activity, with 60 min of moderate-to-vigorous intensity. The “Observatory and Intervention Program of physical exercise and family lifestyles for boys and girls from 4 to 5 years of age in Primary Care” study explores the possibility of improving these recommendations through an online web-based gamified exercise platform in a family setting. The primary objective of this research is to improve anthropometric measurements and physical fitness through increased physical activity. The protocol is expected, based on the knowledge and cutting-edge developments provided by different researchers, to offer an exercise intervention that provides a familiar and easy-to-follow environment for the participants.

The results of this study are expected to provide valuable insights into the impact of multicomponent exercise programs on various health and fitness parameters and inform future guidelines for promoting physical activity in children who do not meet the WHO’s recommendations.

As previously stated, we anticipate that the results obtained by this study will advise future guidelines on the importance of an exercise program for children, especially those who do not meet the WHO recommendations.

## Trial status

Recruitment started in November 2022. Initially, recruitment of participants is expected to be completed by December 2023.

## Data Availability

We will publish the participant-level, de-identified data used in a publicly available online data repository within 1 year of study conclusion. The supplementary material of the final manuscript will contain the full protocol, deidentified dataset, and statistical code. Data used to analyze subgroup sample are available upon reasonable request to Pis BE and AA.

## Abbreviations

BMC: Basic motor competencies
BMI: Body mass index
CRF: Cardiorespiratory fitness
CSBQ: Child Self-Regulation & Behaviour Questionnaire
GPAQ: Global Physical Activity Questionnaire
HGS: Handgrip strength
MOBAK-KG: Motorsiche Basiskompetenzen Kindergarden
OM: Object movement
PA: Physical activity
PF: Physical fitness
SB: Sedentary behaviors
SDQ: Skills and Difficulties Questionnaire
SLJ: Standing long jump
SM: Self-movement
WC: Waist circumference
WHO: World Health Organization
YAP-S: Youth Activity Profile-Spain
